# System for Fabrication of Large-Area Roll Molds by Step-and-Repeat Liquid Transfer Imprint Lithography

**DOI:** 10.3390/ma13081938

**Published:** 2020-04-20

**Authors:** Hyungjun Lim, Sanghee Jung, Junhyoung Ahn, Kee-Bong Choi, Geehong Kim, Soongeun Kwon, Jaejong Lee

**Affiliations:** 1 Nano-Convergence Mechanical Systems Research Division, Korea Institute of Machinery and Materials, 156 Gajeongbuk-ro, Yuseong-gu, Daejeon 34103, Korea; ajh@kimm.re.kr (J.A.); kbchoi@kimm.re.kr (K.-B.C.); geehong@kimm.re.kr (G.K.); sgkwon@kimm.re.kr (S.K.); 2 Department of Nano-Mechatronics, University of Science and Technology, 217 Gajeong-ro, Yuseong-gu, Daejeon 34113, Korea; 3 Office of Nano-Convergence Technology, National NanoFab Center, 291 Daehak-ro, Yuseong-gu, Daejeon 34141, Korea; jsh82@nnfc.re.kr

**Keywords:** liquid transfer imprint lithography, nanoimprint lithography, nanopattern, roll

## Abstract

The effective production of nanopatterned films generally requires a nanopatterned roll mold with a large area. We report on a novel system to fabricate large-area roll molds by recombination of smaller patterned areas in a step-and-repeat imprint lithography process. The process is accomplished in a method similar to liquid transfer imprint lithography (LTIL). The stamp roll with a smaller area takes up the liquid resist by splitting from a donor substrate or a donor roll. The resist is then transferred from a stamp roll to an acceptor roll and stitched together in a longitudinal and, if necessary, in a circumferential direction. During transfer, the nanostructured resist is UV-exposed and crosslinked directly on the acceptor roll. The acceptor roll with the stitched and recombined stamp patterns is ready to be used as a large-area roll mold for roll-based imprinting. A system for this purpose was designed, and its operation was demonstrated taking the example of an acceptor roll of 1 m length and 250 mm diameter, which was covered by 56 patterned areas. Such a system represents an elegant and efficient tool to recombine small patterned areas directly on a large roll mold and opens the way for large-area roll-based processing.

## 1. Introduction

Roll-based nanoimprint lithography (Roll-NIL) has been studied and developed for the fabrication of nanopatterns on large and flexible film substrates. The fabrication of a roll with nanopatterns formed on its surface should be done beforehand in the Roll-NIL process. Most Roll-NIL systems use a roll wrapped by a thin nanopatterned stamp or a nanopatterned belt [1,2,3,4,5,6,7,8,9,10,11]. As the size of the film substrate increases, the nanopatterned roll necessarily becomes longer. However, it is difficult to fabricate nanopatterns onto such a large-area roll. First, because most patterning processes were developed with wafer substrates, it is challenging to apply these processes to roll surfaces. Second, the resist cannot be coated onto the roll via a spin-coater. The dip-coating [12,13,14] and spray-coating [15,16] of the resist on the roll surface were studied as alternatives to spin coating. However, it is difficult to dip a large-area roll with a length of a few hundreds of mm into a resist container. Although the resist is successfully coated on the large-area roll surface, the properties of the resist may change during the process since the process time for the large-area is relatively longer than that for a single wafer substrate.

We designed and demonstrated a novel system to solve the aforementioned problem of coating a large-area roll with nanopatterns. The system can fabricate patterned large-area roll molds by recombination of smaller patterned areas in a step-and-repeat imprint lithography process. The resist is transferred from a stamp roll to an acceptor roll and stitched together in a longitudinal and, if necessary, in a circumferential direction. The transfer is accomplished in a way that is similar to liquid transfer imprint lithography (LTIL), which was originally developed for flat substrates [17,18,19,20,21]. The stamp roll with a smaller area takes up the liquid resist by splitting from a donor substrate or a donor roll, and then the resist is transferred to an acceptor roll.

## 2. Process

### 2.1. Process Design for Fabrication Large-Area Roll Molds

For the LTIL process, three components are required: a stamp, a donor substrate and an acceptor substrate. After coating the resist onto the donor, the stamp takes approximately half of the resist from the donor through contact and separation. The resist on the stamp can then be transferred onto the acceptor. During the process of transferring the resist to the acceptor, it forms an inverse shape with regard to the patterns of the stamp [5].

**Figure 1:**
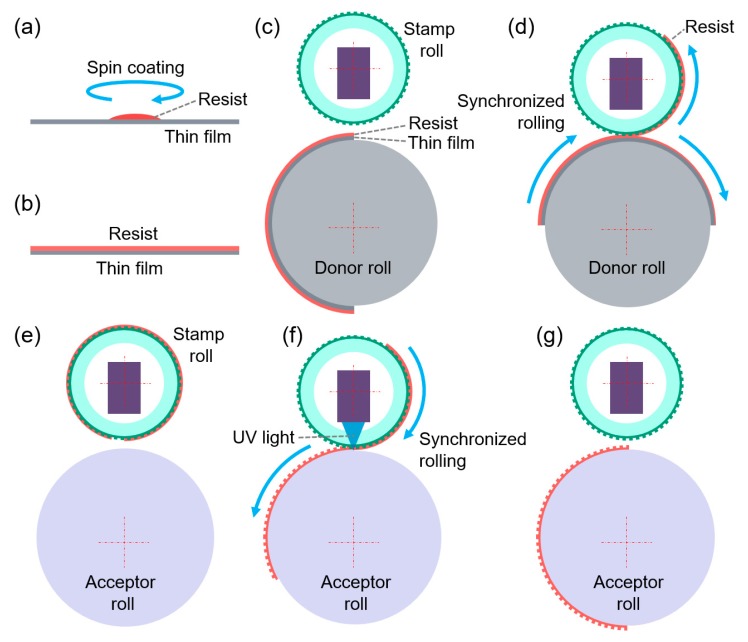
The process for fabrication of large-area roll molds by liquid transfer imprint lithography. (**a**) Resist is dispensed on a thin film, and the film is spun to make uniform thin coating; (**b**) the resist-coated thin film; (**c**) the resist-coated thin film is wrapped onto the donor roll; (**d**) the stamp roll takes up the resist by splitting from a donor roll; (**e**) the stamp roll with the resist is moved to the acceptor roll; (**f**) the resist of the stamp roll is transferred to an acceptor roll while the two rolls rotate synchronously and UV light illuminates; (**g**) a single step of the liquid transfer imprint lithography is finished.

We have modified the flat-based LTIL process to be suitable for making large rollers, as shown in Figure 1. The proposed process uses the stamp, the acceptor and the donor, which are all cylindrical in shape, and their longitudinal axes are positioned such that they are parallel. The contact area therefore forms a narrow line. Since the donor is cylindrical, the resist can be coated onto the cylindrical surface of the donor by means of dip-coating or spraying. However, in the actual work, a resist-coated thin film in this work is wrapped onto the donor. (Figure 1a–c). The stamp roll, having a cylindrical shape, rolls on the donor to transfer about half of the resist from the donor to the stamp roll. (Figure 1d)

The patterns as well as the resist material of the stamp roll can then be transferred to the acceptor, as shown in Figure 1f. After the resist-coated stamp roll is aligned to one side of the acceptor roll in parallel, two of them can be put into contact and pressed by moving the stamp roll toward to the acceptor roll. The synchronous rotation of two rolls creates line-by-line contact between the stamp roll and the acceptor roll. The UV source inside the stamp roll illuminates UV light onto the contact area. The resist on the stamp roll can be cured and transferred to the acceptor roll by UV light irradiation.

### 2.2. Step-and-Repeat Process

**Figure 2:**
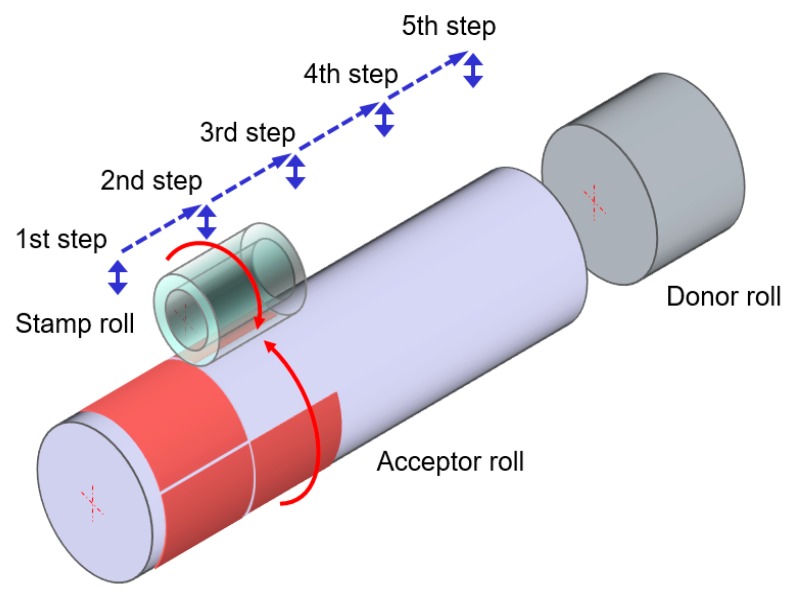
Step-and-repeat manner in the longitudinal direction of the proposed process.

If the diameter of the acceptor roll is greater than that of the stamp roll, the process can be likewise repeated in the circumferential direction [22]. If the acceptor roll is longer than the stamp roll, this process should be also repeated in the longitudinal direction, as shown in Figure 2. The rotational and axial position of the stamp roll relative to the acceptor roll should be measured and controlled to minimize stitching and seaming errors. This calculation also requires the actual radii of both rolls.

## 3. Experiments

### 3.1. System

**Figure 3:**
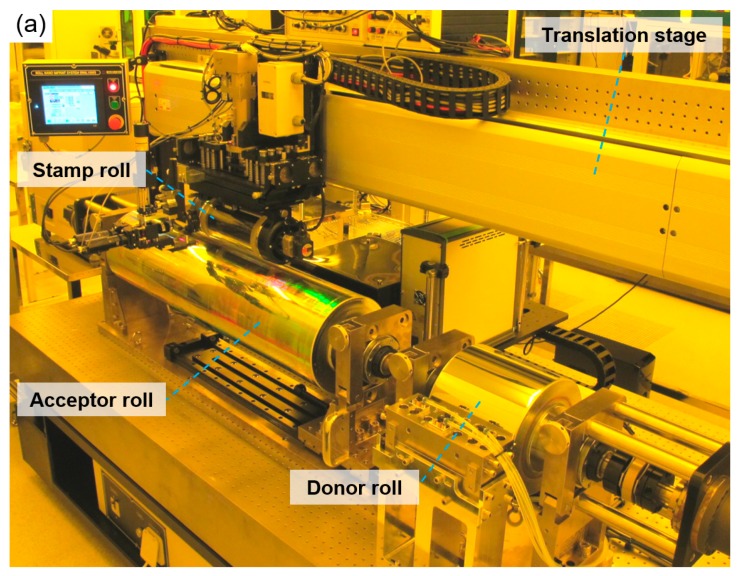

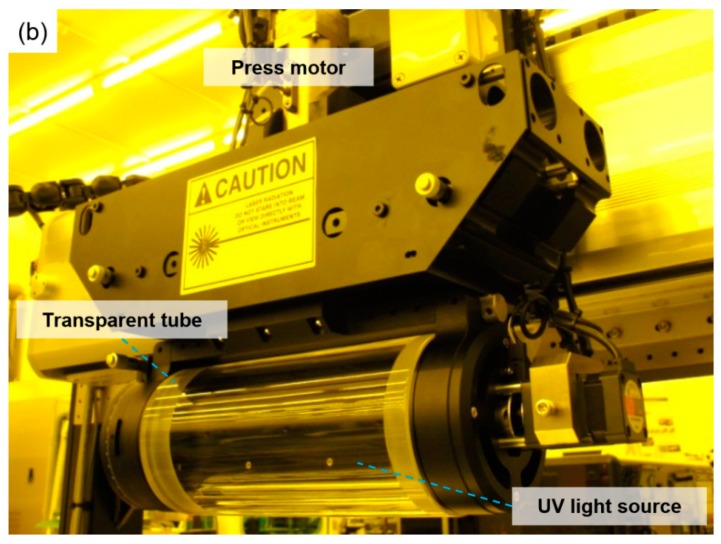
Pictures of the developed system for fabrication of large-area roll molds. (**a**) System overview; (**b**) the stamp roll unit.

A roll-based system was developed to demonstrate the proposed process, as shown in Figure 3a. An acceptor roll with a maximum length of 1016 mm and a diameter of 250 mm can be loaded onto and unloaded from the system. A donor roll with a length of 216 mm and a diameter of 250 mm is also loaded onto the system. The center of the donor roll is aligned to coincide with the center of the acceptor roll. The acceptor and donor rolls are rotated by their rotational motors, respectively.

There is a stamp roll above the acceptor and donor rolls, as shown in Figure 3b. The stamp roll consists of a transparent quartz tube (Daeyang Optical Co., Daejeon, Korea) with an effective length of 200 mm. The tube has an outer diameter of 125 mm. The tube is changeable by simply separating one side of its rotational guide. Therefore, the tube can be standardized, similar to the wafer used in the flat process. 

The stamp roll can move in the horizontal direction by the translation stage for the step-and-repeat process. It can also move to be positioned above the donor roll to take the resist from the donor roll for every single step of the process. In the developed system, the step-and-repeat process does not measure or correct the position of the stamp and the substrate by using a measuring tools such as cameras. However, after moving at a specified step interval, a subsequent process was performed. The stroke of the translation stage of the stamp roll is 1500 mm, the resolution is 0.4 μm, the repeatability is 20 μm and the straightness is within 5 μm. After the translational movement, the stage holds the stamp roll unit firmly during a single process.

The stamp roll can be moved up and down by a press actuator using a servo motor (Panasonic Co., Osaka, Japan) so that the stamp roll comes into contact with the donor roll and the substrate roll under constant load conditions. The linear actuator has a stroke of up to 100 mm, can be driven at a speed of up to 2 mm/s, has a resolution of 0.1 μm and has a rated load of 2.94 kN. For precise control of the contact force, several coil springs were mounted between the press motor flange and the stamp roll holder to configure the elastic modulus to be 98 N/mm. The actual applied force is measured by using load cells arranged in a triangle inside the stamp roll holder, and control is performed to control the drive of the press motor to be a constant force. While maintaining the contact condition, the stamp roll and the acceptor roll can be synchronously rotated by their rotational motors and the controller.

**Figure 4:**
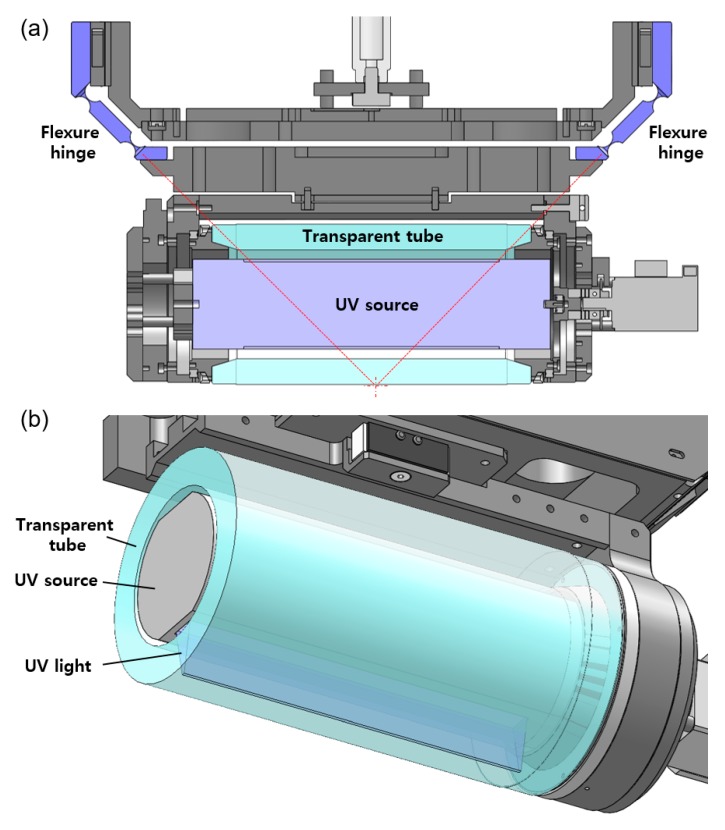
Cross section images of the stamp roll unit. (**a**) The passive angle compensation mechanism using flexure hinges; (**b**) the UV source installed inside the stamp roll (transparent tube).

When the stamp roll contacts the donor roll or acceptor roll, the load distribution of the contacting line may not be uniform. The main reason is that the axis of the stamp roll is not parallel to the axis of other rolls. As shown in Figure 4a, a passive angle compensation mechanism using flexure hinges was applied inside the stamp roll unit. When compensating for the angle difference, the center of rotation was designed to come to the surface of the stamp to prevent slippage between the stamp roll and the donor roll or acceptor roll. 

The UV source is installed inside of the stamp roll (transparent tube) as shown in Figure 4b. It can illuminate a line of focused UV light by 36 LEDs (Nichia Co., Tokushima, Japan) of 365 nm wavelength and a cylindrical lens. The length and width of the linearly focused UV light are 206 and 2 mm, respectively. The maximum optical power is 20 mW/cm^2^.

### 3.2. Patterning

**Figure 5:**
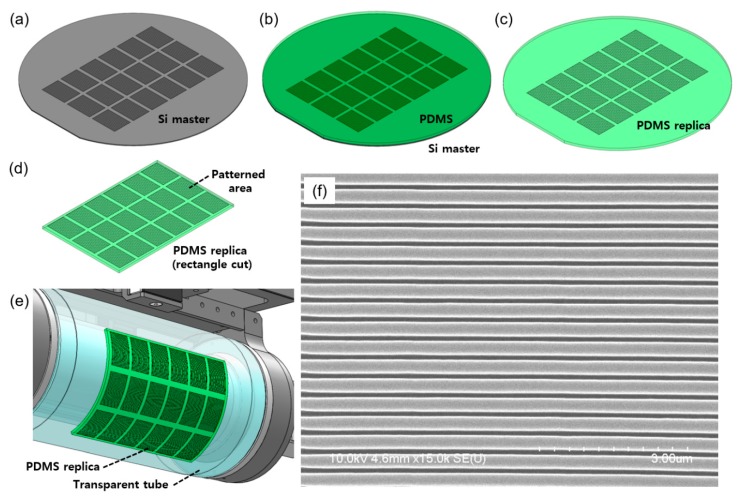
Fabrication of the PDMS stamp roll. (**a**) A nanopatterned Si template; (**b**) PDMS poured on the Si template; (**c**) PDMS replica; (**d**) rectangle cut PDMS replica; (**e**) the PDMS replica mounted on the transparent tube; (**f**) SEM image of the nanolines on the PDMS replica.

The nanopatterns can be directly fabricated on the surface of a transparent tube, or a nanopatterned film can be attached to the surface of the tube to prepare the stamp roll. For this experiment, we first fabricated a polydimethylsiloxane (PDMS) replica from a nanopatterned silicon (Si) template as shown in Figure 5a–d. The PDMS was made by mixing a 10:1 ratio of Sylgard 184 base and Sylgard 184 cure agent (Dow Corning Co., Midland, MI, USA). After the mixture was poured onto the Si template (Figure 5b), it was degassed in a vacuum desiccator for 10 min and cured at 70 °C in an oven for 5 h. The thickness and Young’s modulus of the PDMS replica were 2.5 mm and 1.48 MPa, respectively. The PDMS replica should be uniform in thickness to produce conformal contact with the acceptor roll during this process. We cut it into rectangles of 144 mm by 96 mm in size (Figure 5d) and mounted to the surface of the transparent tube. PDMS adheres well to the quartz surface, so after attaching it to the transparent tube, the edges were fixed with adhesive tape to prevent lifting. There are nanolines, as shown in Figure 5f, with a width, pitch and depth of 150, 400 and 300 nm, respectively, in the patterned area of the PDMS replica. 

A thin polyethylene terephthalate (PET) film 0.2 mm thick and 150 mm by 150 mm in size was used as a thin film. The polyurethane-acrylate (PUA, NINS-511RM, Changsung sheet, Cheonan, Korea) resist mixed with a solvent was spin-coated onto the thin film at the speed of 3000 rpm for 30 s. We adjusted the ratio of the solvent to get the thickness of 600 nm. The thin film was attached to the surface of the donor roll with adhesive tape within 120 s after the coating was finished. Then, the stamp roll came into contact with the donor roll under a load of 49 N, and both rolls synchronously rotated with a linear velocity of 0.4 mm/s. Half of the resist was then transferred from the donor film to the surface of the stamp roll. Some of the resist was not reliably transferred when the linear velocity was faster than 0.4 mm/s.

**Figure 6:**
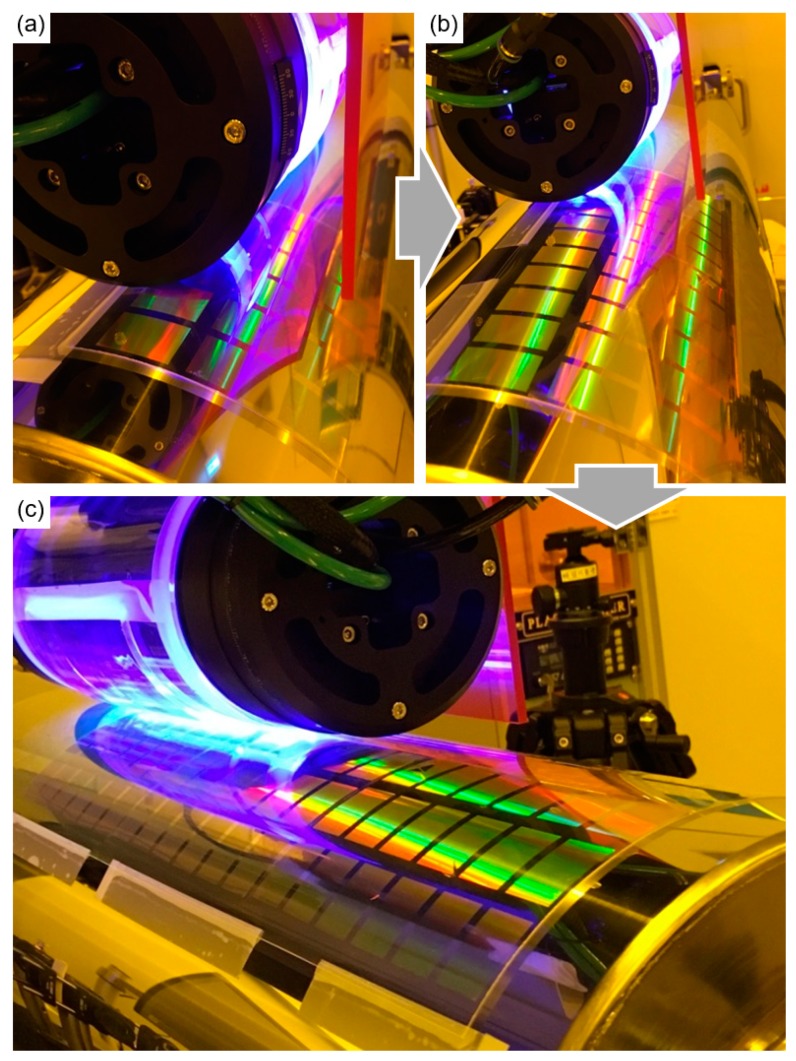
Photographs of the system and the patterned acceptor roll during (**a**) the first, (**b**) second and (**c**) third steps of the transfer process in longitudinal direction under the UV light illumination.

The acceptor roll is made of steel, and a 70 μm thick PET film is wrapped around it with adhesive tape. As the next step in the single process, the stamp roll was moved to a designed position of the acceptor roll. The two rolls were then pressed and synchronously rotated with a linear velocity of 0.12 mm/s, which satisfies the curing condition of the resist, as shown in Figure 6. The resist was then transferred from the stamp roll to the acceptor roll, forming an inverse shape relative to the stamp roll pattern. 

We performed a total of 56 processes under the same conditions repeatedly for all areas of the acceptor roll. Each process was allowed to proceed with a distance of 1 mm from the previous process. Thus, the stamp roll was moved 145 mm in the longitudinal direction and 97 mm in the circumferential direction of the acceptor roll for the next process.

## 4. Discussion

**Figure 7:**
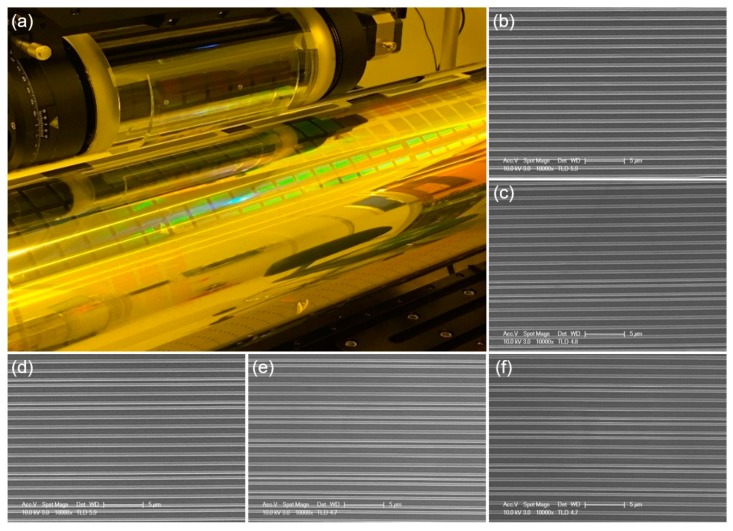
Photograph and SEM images of the patterned results on a large-area acceptor roll after 56 processes have been finished. (**a**) Photograph of the acceptor roll and the stamp roll; (**b**–**f**) SEM images measured at random locations on the PET film peeled off the acceptor roll. The lines are mounted in circumferential direction.

The patterned acceptor roll after the step-and-repeat process is shown in Figure 7a. The transferred nanopatterns (vertical lines) were measured via scanning electron microscopy (SEM), as shown in Figure 7b–f. Since the acceptor roll was covered with a PET film, the film was peeled off and an arbitrary position was selected to measure after the experiment. The SEM image shows that the patterns on the acceptor roll have the inverse shape of that of the PDMS replica on the stamp roll. Some of the lines have irregular pitch, which is thought to be due to the pattern being transferred while the PDMS stamp is deformed. Therefore, it is necessary to precisely adjust the parameters such as performing the process with a load as low as possible among the conditions in which the resist is cured and transferred.

In the patterning process using ultraviolet curing, there is always a proximity effect due to ultraviolet light leaking due to diffraction of light [22]. If the resist is coated on all areas of the large-area substrate and imprinted in a step-and-repeat method, patterning is performed on an area of the substrate equal to the stamp area after one process, but the nearby resist is also cured due to ultraviolet light leaking around the stamp. Therefore, when performing the next process, there is a problem in that pattern formation is not performed well at the boundary that has already completed the process. This phenomenon is the most serious problem in reducing the stitching error. When fabricating a stamp, there are many restrictions, such as blocking the transmission of ultraviolet light outside the pattern area. The process using LTIL technology proposed in this paper is a method to fundamentally eliminate the effects of the proximity effect. One process does not affect the next process because the resist is not coated on the entire surface of the substrate and is only transferred from the stamp through each process.

## 5. Conclusions

We designed, constructed and verified a novel system to fabricate large-area roll molds by a step-and-repeat imprint lithography process similar to LTIL. The small roll, a transparent tube with an effective length of 200 mm and a diameter of 125 mm, was installed as a stamp roll. Nanopatterns can be directly fabricated onto the surface of the stamp roll. However, we wrapped nanopatterned PDMS sheets with a size of 144 mm by 96 mm around the transparent tube of a diameter of 125 mm for the stamp roll. A 0.2 mm thick PET film was spin-coated with a resist and attached to a donor roll of a diameter of 250 mm. The large roll, a metal roll with an effective length ranging from 500 to 1000 mm and a diameter of 250 mm, was mounted as an acceptor roll. 

The stamp roll took up the resist by splitting from the donor roll, and then the resist was transferred while being cured from the stamp roll to the acceptor roll. Eventually, nanopatterns with a width of 150 nm were transferred to the acceptor roll of a length of 1 m by repeating this process 56 times. We tried to find the conditions by performing various processes on a single substrate. In particular, a PET film was attached to the surface of the polished metal roll to facilitate measurement. Since this paper focuses on verifying that the process based on LTIL performs well from one roll surface to another, the results of the experiment cannot be used directly for the subsequent processes such as the etching or electroplating. 

To fabricate nanopatterns of metal onto the acceptor roll, this process should be directly conducted on the metal surface of the acceptor roll without residual of the resist, and a subsequent processes should then be performed. The first step in future work will therefore be to verify the process on a metal surface and to find resist material which can be easily removed. However, the developed system can be a good tool to recombine small patterned areas directly on a large roll mold, which could be employed in large-area roll-based processes for the mass production of functional films.

## References

[1] Ahn S.H., Guo L.J. (2009). Large-area roll-to-roll and roll-to-plate nanoimprint lithography: A step toward high-throughput application of continuous nanoimprinting. ACS Nano.

[2] Han J., Choi S., Lim J., Lee B.S., Kang S. (2009). Fabrication of transparent conductive tracks and patterns on flexible substrate using a continuous UV roll imprint lithography. J. Phys. D Appl. Phys..

[3] Nagato K., Sugimoto S., Hamaguchi T., Nakao M. (2010). Iterative roller imprint of multilayered nanostructures. Microelectron. Eng..

[4] Dumond J.J., Mahabadi K.A., Yee Y.S., Tan C., Fuh J.Y.H., Lee H.P., Low H.Y. (2012). High resolution UV roll-to-roll nanoimprinting of resin moulds and subsequent replication via thermal nanoimprint lithography. Nanotechnology.

[5] Ok J.G., Youn H.S., Kwak M.K., Lee K.T., Shin Y.J., Guo L.J., Greenwald A., Liu Y. (2012). Continuous and scalable fabrication of flexible metamaterial films via roll-to-roll nanoimprint process for broadband plasmonic infrared filters. Appl. Phys. Lett..

[6] Yoshikawa H., Taniguchi J., Tazaki G., Zento T. (2013). Fabrication of high-aspect-ratio pattern via high throughput roll-to-roll ultraviolet nanoimprint lithography. Microelectron. Eng..

[7] Jiang M., Lin S., Jiang W., Pan N. (2014). Hot embossing holographic images in BOPP shrink films throughlarge-area roll-to-roll nanoimprint lithography. Appl. Surf. Sci..

[8] Mäkelä T., Kainlauri M., Willberg-Keyriläinen P., Tammelin T., Forsström U. (2016). Fabrication of micropillars on nanocellulose films using a roll-to-roll nanoimprinting method. Microelectron. Eng..

[9] Leitgeb M., Nees D., Ruttloff S., Palfinger U., Götz J., Liska R., Belegratis M.R., Stadlober B. (2016). Multilength Scale Patterning of Functional Layers by Roll-to-Roll Ultraviolet-Light-Assisted Nanoimprint Lithography. ACS Nano.

[10] Retolaza A., Juarros A., Ramiro J., Merino S. (2018). Thermal roll to roll nanoimprint lithography for micropillars fabrication on thermoplastics. Microelectron. Eng..

[11] Lee S.-B., Ji S., Lee J.-Y., Lee J., Yeo J.-S. (2019). Fluorinated low-viscosity copolymer with enhanced release property for roll-to-plate UV nanoimprint lithography. Nanotechnology.

[12] Taniguchi J., Aratani M. (2009). Fabrication of a seamless roll mold by direct writing with an electron beam on a rotating cylindrical substrate. J. Vac. Sci. Technol. B.

[13] Maruyama H., Unno N., Taniguchi J. (2012). Fabrication of roll mold using electron-beam direct writing and metal lift-off process. Microelectron. Eng..

[14] Huang K.-F., Lee Y.-C. (2013). Fabrication of metal roller mold with submicrometer feature size using contact printing photolithography technique. J. Vac. Sci. Technol. B Nanotechnol. Microelectron..

[15] Tsai S.-W., Chen P.-Y., Lee Y.-C. (2014). Fabrication of a seamless roller mold with wavy microstructures using mask-less curved surface beam pen lithography. J. Micromech. Microeng..

[16] Tahir U., Kamran M.A., Jang M.H., Jeong M.Y. (2019). Thin-film coating on cylinder for fabrication of cylindrical mold: Roll-to-roll nano-imprint lithography. Microelectron. Eng..

[17] Koo N., Kim J.W., Otto M., Moormann C., Kurz H. (2011). Liquid transfer imprint lithography: A new route to residual layer thickness control. J. Vac. Sci. Technol. B.

[18] Lee J., Park H., Choi K., Kim G., Lim H. (2014). Fabrication of hybrid structures using UV roll-typed liquid transfer imprint lithography for large areas. Microelectron. Eng..

[19] Moro M., Taniguchi J. (2015). Removal of residual layer by liquid transfer imprint lithography using roll-to-roll UV-NIL. Microelectron. Eng..

[20] Uchida T., Yu F., Nihei M., Taniguchi J. (2016). Fabrication of antireflection structures on the surface of optical lenses by using a liquid transfer imprint technique. Microelectron. Eng..

[21] Unno N., Kigami H., Fujinami T., Nakata S., Satake S., Taniguchi J. (2017). Fabrication of calibration plate for total internal reflection fluorescence microscopy using roll-type liquid transfer imprint lithography. Microelectron. Eng..

[22] Lim H., Choi K., Kim G., Lee S., Park H., Ryu J., Jung S., Lee J. (2014). Roll-to-roll nanoimprint lithography for patterning on a large-area substrate roll. Microelectron. Eng..

